# Parent artery reconstruction for large or giant cerebral aneurysms using a Tubridge flow diverter (PARAT): study protocol for a multicenter, randomized, controlled clinical trial

**DOI:** 10.1186/1471-2377-14-97

**Published:** 2014-05-04

**Authors:** Yu Zhou, Peng-Fei Yang, Yi-Bin Fang, Yi Xu, Bo Hong, Wen-Yuan Zhao, Qiang Li, Rui Zhao, Qing-Hai Huang, Jian-Min Liu

**Affiliations:** 1 Department of Neurosurgery, Changhai Hospital, Second Military Medical University, 168 Changhai Road, Shanghai 200433, P.R. China

**Keywords:** Large, Giant, Aneurysm, Tubridge, Flow diverter, Trail

## Abstract

**Background:**

The treatment of large (10-25 mm) or giant (≥25 mm) cerebral aneurysms remains technically challenging, with a much higher complication and recanalization rate than that is observed for smaller aneurysms. The use of a flow diverter seems to facilitate the treatment of this special entity. In a previous single-center prospective study approved by the Ethics Committee and China Food and Drug Administration (CFDA), we obtained promising results, showing remarkable safety and effectiveness for the Tubridge flow diverter. Nevertheless, the previous study may have been limited by biases due to its single-center design and limited number of subjects. Furthermore, although various articles have reported durable results from treating aneurysms using flow diverters, increasing questions have arisen about this form of treatment. Thus, prospective, multiple-center, randomized trials containing more subjects are needed.

**Methods/Design:**

This study is a multicenter, randomized, controlled clinical trial comparing clinical outcomes for patients with unruptured large/giant intracranial aneurysms treated with either conventional stent-assisted coiling or flow diverter implantation. A total of 124 patients who fulfill the inclusion and exclusion criteria will be randomized into either a treatment group or a control group in the ratio of 1:1. The treatment group will receive Tubridge implantation alone or combined with bared coils, and the control group will be treated with stent-assisted coiling (bare coils). The primary endpoint will be the complete occlusion rate at 6-month follow-up. Secondary endpoints include the immediate technique success rate, overall mortality, adverse events (ischemic stroke or intracranial bleeding) within 30 days, 90 days and 1 year post-operation, and the rate of intra-stent stenosis and thrombosis 6 months post-operation.

**Discussion:**

This prospective trial may provide more information on the safety and efficacy of the Tubridge flow diverter and may potentially change the strategy for treatment of large or giant aneurysms.

**Trial registration:**

The trial is registered on the Chinese Clinical Trial Registry: ChiCTR-TRC-13003127

## Background

Intracranial aneurysms are pathological bulges of cerebral arteries caused by various etiologies, such as inflammation or hemodynamic stress. Their rupture often results in a catastrophic outcome, and they are associated with high rates of mortality and morbidity [1]. Large (10-25 mm) or giant (≥25 mm) aneurysms are a subtype of intracranial aneurysm with a much higher rupture risk and poorer outcomes compared with small aneurysms [2,3]. Despite technical advances, the treatment of large or giant aneurysms remains technically challenging, with a much higher complication and/or recanalization rate [4-8].

In recent years, with better understanding of the occurrence and development of intracranial aneurysms, the concept of vessel reconstruction, especially flow diversion, has attracted the interest of neurointerventionalists worldwide. Accordingly, various flow diversion devices have emerged around the world; the best known devices include the Pipeline (ev3, Irvine, CA, USA) and the Silk (Balt Extrusion, Montmorency, France), which gained the approval of the FDA and the CE and have been used clinically in over 50 countries [9-16].

Compared with conventional endovascular treatment such as stent-assisted coiling, the Flow Diverter (FD) can alter the blood flow, reduce the hemodynamic stress in the aneurysm neck, and promote intra-saccular thrombus formation [17-20]. A series of studies have shown that flow diverters can significantly improve the outcome of intracranial aneurysms [9-11,13,14,16].

The Tubridge is a new type of flow diversion device developed by MicroPort Medical Company (Shanghai, China) based on our previous clinical experience of multiple stenting in intracranial aneurysms and the hemodynamic study of intracranial aneurysms. Referring to its structure, the Tubridge FD is actually a braided, self-expanding “stent-like” device with flared ends. Currently, Tubridge FDs are available in many diameters (2.5-6.5 mm) and lengths (12–45 mm). A large Tubridge (diameter ≥3.5 mm) is braided with 62 nickel- titanium microfilaments and 2 platinum-iridium radiopaque microfilaments, whereas a smaller Tubridge (diameter <3.5 mm) is composed of 46 nitinol and 2 platinum-iridium microfilaments. All Tubridge FDs were designed with a pore size of 0.040-0.050 mm^2^ at a nominal diameter, aiming to provide a high metal coverage (approximately 30.0%-35.0%) at the aneurysmal neck after full opening. In addition to its use of a nickel-titanium alloy (commonly known as nitinol, which has the advantages of shape-holding memory and super-elasticity) and flared ends, the Tubridge FD offers some structural improvements compared with the Pipeline and the Silk. The use of platinum-iridium material for the radiopaque microfilaments allows for improved visualization of both diameter and length during the procedure. More importantly, the design, which includes more braided microfilaments for large-size FDs, decreases the shortening rate of the FD after its full opening.

In a previous single-center prospective study approved by the Ethics Committee and China Food and Drug Administration (CFDA), we obtained promising results, showing remarkable safety and effectiveness for the Tubridge FD. In that study(data in press), a total of 33 Tubridge FDs were successfully implanted for 28 large or giant aneurysms, with the exception of one mid-stent with a poor opening, resulting in a technical success rate of 97.0% (32/33). Follow-up angiographies, taken at a mean follow-up of 9.9 m (5–24 m), were available for 25 aneurysms. Of those, 18 (72.0%) aneurysms were completely occluded, 6 (24.0%) were improved, and 1 (4.0%) was unchanged. All visible covered branches and the parent artery were patent, with no stenosis or obliteration. During a follow-up period of 6 to 30 months (mean 19.0 months), the symptoms experienced were resolved in 13 patients, improved in 6, and showed no change in 4. No procedure-related morbidity or mortality occurred. Nevertheless, biases may exist in the study due to its single-center design and limited number of subjects. Prospective, multiple-center, randomized trials that include more subjects are needed.

Furthermore, although various articles have reported durable results from treating aneurysms with FDs, increasing questions have arisen about this type of treatment. For example, is the occurrence of peri-procedure intra-parenchymal hemorrhage accidental or related to the application of the device? Are ischemic complications encountered more often with FDs than with conventional therapy [21-25]? These questions need to be answered through prospective trials, which have been lacking to date.

Based on these considerations, we initiated this trial with the aim of evaluating the safety and effectiveness of the Tubridge FD for unruptured large or giant aneurysms (≥10 mm).

## Methods

### Objectives

The aim of the trial is to assess the safety and effectiveness of the Tubridge flow diverter in the treatment of unruptured large or giant intracranial saccular aneurysms.

### Study design

This study is a multicenter, randomized, controlled clinical trial comparing clinical outcomes for patients with unruptured large/giant intracranial aneurysms treated with either conventional stent-assisted coiling or with flow diverter implantation. The study will be conducted in 13 centers, each of which have performed more than 30 stent-assisted coiling procedures per year in the past 5 years, after gaining approval from local ethics committees. A total of 124 patients who fulfill the inclusion and exclusion criteria will be randomized into either a treatment group or a control group in the ratio of 1:1. The treatment group will receive Tubridge FD implantation alone or combined with bare coils, and the control group will receive stent-assisted coiling (bare coils). Procedure-related complications, adverse events, and follow-up data will be collected prospectively to evaluate the safety and effectiveness of the Tubridge FDs.

### Hypothesis

Compared with conventional stent-assisted coiling, Tubridge FD implantation does not enhance the risk of procedure-related complications and may lead to a higher complete occlusion rate and a decreased recanalization rate.

### Primary and secondary outcomes

The primary endpoint will be the complete occlusion rate at 6-month follow-up.

Secondary endpoints will include:

1. Immediate technique success rate.

2. Overall morbidity or mortality (assessment time point: 30 days, 90 days and 1 year post-operation).

3. Ischemic stroke or intracranial bleeding (assessment time point: 30 days, 90 days and 1 year post-operation)

4. The rate of intra-stent stenosis (assessment time point: 6 months post-operation)

5. The rate of intra-stent thrombosis (assessment time point: 6 months post-operation)

6. General adverse events (assessment time point: 30 days, 90 days and 1 year post-operation).

### Participants

#### *Inclusion criteria*

Patients eligible for inclusion will meet the following criteria:

1. Age 18 years to 75 years, male or nonpregnant female.

2. Unruptured internal carotid artery or vertebral artery saccular aneurysm (including recanalized aneurysms), confirmed by CTA, MRA, or DSA.

3. Width of aneurysm neck ≥4 mm, and aneurysm size ≥10 mm.

4. Parent vessel with a diameter range of 2.0-6.5 mm.

5. Indications for Enterprise stent-assisted coiling and Tubridge FD implantation with or without coiling.

6. Is willing to return to the investigational site for follow-up according to our protocol.

7. Understands the nature of the procedure and provision of written informed consent.

#### *Exclusion criteria*

Patients will be excluded if they meet any of the following criteria:

1. No appropriate route to access the aneurysm via endovascular approach.

2. A non-treated arteriovenous malformation (AVM) in the territory of the target aneurysm.

3. Ruptured aneurysms.

4. Anatomy not appropriate for endovascular treatment due to severe intracranial vessel tortuosity or stenosis, or intracranial vasospasm not responsive to medical therapy.

5. Major surgery within the previous 30 days or planned within the next 90 days after the enrollment date.

6. Severe neurological deficit that renders the patient incapable of living independently (modified Rankin score ≥4).

7. Co-morbid conditions that may limit survival to less than one year.

8. Enrollment in another trial.

9. Poor compliance (inability to complete the full study).

10. Allergy or contraindication for the use of nickel-titanium alloy.

11. Inability to receive anti-platelet or anticoagulant medication.

12. History of life-threatening allergy to contrast dye.

#### *Withdrawal criteria*

Patients who have been included in this trial and have given written informed consent but are not able to complete the whole study for any reason will be withdrawn, including circumstances as below:

1. The subjects voluntarily quit the trial for various reasons.

2. Occurrence of serious adverse events. The subjects, main researchers, ethics committee, supervisor or CFDA terminate the research based on the consideration of ethics.

3. Early termination of the process based on the investigator’s judgment (to prevent development of severe complications, and so on).

4. Significant deviation in implementation, or the subject failed to comply with the protocol.

5. Lost of follow-up due to changes in working/living places, or fortuitous accident. When these fortuitous accidents occurs, including traffic accident, death,or bone fracture, et al., Close follow-up should be conducted to determine their relationship with the usage of Tubridge FD.

6. flawed or absence of informed consents.

When the subjects are withdrawn, the researcher would try their best to contact the subjects via telephone call, letter, clinic interview, et al., and document the reasons why they are withdrawn in detail; of which, those experienced adverse effects must be recorded in the CRF if the adverse effects were thought related to the devices used in this trial. All raw data of those enrolled and treated subjects must be kept including subjects withdrawn.

### Method of allocation and randomization

All recruited subjects will be randomly allocated to either the flow diverter implantation group or the control group using a web-based platform linked to a central randomization database. According to existing research, aneurysms larger than 15 mm with posterior circulation are thought to carry a higher complication rate [26,27]. Stratification will be performed for aneurysm size (≦15 mm versus >15 mm) and aneurysm location (anterior circulation versus posterior circulation).

### Sample size

The sample size calculation is based on the study of Mocco et al. [28], who reported the midterm results of 219 patients treated with Enterprise-assisted stent coiling. In their research, the 6-month complete occlusion rate was 40%; of these, 46.4% were cases of aneurisms smaller than 7 mm. Because the aneurysmal complete occlusion rate decreases with aneurysm size, we assume the 6-month complete occlusion rate to be 35% following Enterprise stent-assisted coiling for aneurysms larger than 10 mm. Furthermore, the 6-month complete occlusion rate for the Tubridge FD implantation is predicted to be 65% according to our previous results and to those of other studies using Silk or Pipeline implantation. Thus, at least 98 patients will need to be randomized in the ratio of 1:1 (49 in each group) to obtain a positive result (two-tailed test, significance level of α = 0.05, power (1 - β) = 0.80). Considering a withdrawn-patient maximum of 20%, the sample size will increase to at least 124 (62 in each group).

### Procedure

For all recruited patients, dual antiplatelet drugs (300 mg/day aspirin plus 75 mg/day clopidogrel) will be given for at least 3 days before the procedure. All procedures will be performed under general anesthesia and via the transfemoral approach. After sheath placement, heparin will be given to maintain the activated clotting time at 2 to 3 times the baseline throughout the procedure. Next, a proper (7 F) guiding catheter will be placed in the distal internal carotid artery or vertebral artery. Subsequently, the aneurysms will be treated with conventional stent-assisted coiling or FD implantation with or without coiling. All detailed data, such as the aneurysm’s actual size, width of the aneurysmal neck, aneurysmal shape, diameter of the parent artery, and treatment results, will be recorded. The post-operative antiplatelet regimen will be administered as follows: <6 w: 300 mg aspirin + 75 mg clopidogrel; 6 w-3 m: 100 mg aspirin + 75 mg clopidogrel; ≥3 m: 100 mg aspirin indefinitely.

### Planned patient follow-up

Arrangements will be made for all patients to be evaluated at discharge and 30 d (±7 d), 90 d (±15 d), and 1 year (±30 d) post-treatment, either by clinic or telephone contact. Postoperative DSA follow-up is required after 6 m (±30 d).

### Safety and efficacy assessment

During the procedure and at each follow-up time point, information about the patients will be documented in detail. All data will be collected and analyzed by an independent central laboratory to compare the complication rate, morbidity, mortality, as well as initial and follow-up angiographic results between the two groups. If any adverse event has occurred, the detailed symptoms, duration, severity, possible reasons, actions taken, results and other relevant information will be reported. In addition, researchers must fill out a serious adverse event (SAE) form and notify the principal investigator, institutional review board (IRB), China Food and Drug Administration (CFDA), Clinical Research Associate (CRA), and other regulatory authorities within 24 hours.

Immediate angiographic results will be classified according to Raymond’s classification for those treated with stent-assisted coiling or FD implantation combined with coils [29]. For aneurysms treated with the Tubridge FD alone, flow modifications will be defined as disrupted inflow jet, slow flow (if the contrast circulation within the aneurysm is slowed), or reduced contrast filling (if increased contrast stagnation is observed within the aneurysm at the late venous phase of the angiographic series) [30].

The angiographic results will be classified into four categories when compared with the immediate degree of embolization: 1) Occluded, defined as no contrast filling into the aneurysm sac; 2) Improved, defined as decreased contrast filling into the aneurysm sac; 3) Stable, defined as unchanged contrast filling into the aneurysm sac; 4) Recanalized, defined as increased contrast filling into the aneurysm sac. The intra-stent stenosis will also be documented.

Table 1 shows a summary of the intervention and follow up.

**Table 1:** Summary of measurement items and point of data capture

**Point of data capture**	**Pre-procedure**	**During procedure**	**Post-procedure**					
			**24 hours**	**Discharge**	**30 days**	**90 days**	**6 months**	**1 year**
Window phase					±7 d	±15 d	±30 d	±30 d
Informed consent form	√							
Inclusion/exclusion criteria	√							
Withdrawal criteria		√			√	√	√	√
Medical history/demographic information	√							
Physical examination (1)	√			√			√	
Vital signs (2)	√	√		√				
Routine urine and blood test	√			√				
Blood biochemistry test (3)	√			√				
Blood lipid test (4)	√							
Pregnancy tests (5)	√						√	
CTA, MRA or DSA	√							
Clinical evaluation		√			√	√	√	√
DSA examination (6)		√					√	
Adverse events		√	√	√	√	√	√	√

1. Physical examination: height, weight, and neurological presentation.

2. Vital signs: systolic pressure, diastolic pressure and heart rate.

3. Blood biochemistry tests: fasting blood glucose, blood urea nitrogen, TBIL, and serum creatine.

4. Blood lipid tests: total cholesterol, triglycerides, and low density lipoprotein.

5. Pregnancy tests: restricted to women with suspected pregnancy.

6. DSA examination: angiographic images will be sent to an independent laboratory for data analysis.

### Planned analyses

Statistical analyses will be conducted based on an intention-to-treat (ITT) population. Participants who are recruited and treated will be considered the full analysis set (FAS), those who complete treatment and the entire follow-up protocol will be considered the per-protocol set (PPS), and those who are treated and have at least one safety evaluation will be considered the safety set (SS). Last observation carried forward (LOCF) method will be implemented to handle the missing data from withdrawn subjects, who would be included in FAS.

Comparisons of demographic information will first be conducted to measure the balance between the two groups. Then evaluation of primary and secondary outcome will conducted as below:

Primary outcomes:

The comparison of the treatment groups with regard to the primary endpoint will be performed with the data of the Full Analysis Set as well as of the Per Protocol Set. In case the two analyses will lead to different conclusions the result of the analysis of the Per Protocol Set is deciding.

Use superiority test for analysis of primary outcome. The formal test problem is as follows:

Test problem for the complete occlusion rate at 6-month follow-up:

Let πT be the rate of complete occlusion rate of treatment group at 6-month follow-up.

Let πc be the rate of complete occlusion rate of control group at 6-month follow-up.

The superiority margin δ is 0.

Test problem:

H0: πT -πc ≥ 0

H1: πT -πc < 0

α = 0.05 (2-sided)

The alternative hypothesis will not be accepted unless the lower limit of the 95% confidence interval of the difference of occlusion rate lies above the superiority margin.

Secondary outcomes:

Immediate occlusion rate will be conducted in FAS group, while other safety evaluation will be be conducted in SS group. For quantitative data, t-test (for equal variances and normal distribution) or Wilcoxon rank sum test could be applied according to the distribution of the data. The categorical variables will be compared between study arms using Chi-square tests or Fisher's exact tests (if Chi-square test is not valid). Besides, for immediate technique success rate, 95% confidence interval of each groups and difference between groups will be estimated.

The statistical analysis process will be conducted by a statistician at the Institute of Clinical Evaluation affiliated with Beijing University, and the data will be analyzed using Statistical Analysis System (SAS) software version 9.2 (SAS Institute Inc., Cary, NC, USA).

### Ethics approval

Thirteen institutes will take part in this trial, and the trial has been approved by all relevant local ethics boards including Shanghai changhai hospital ethics committee, Huashan hospital ethics committee, Xuanwu hospital ethics committee, Tiantan hospital ethics committee, ethics committee of the second affiliated hospital of Zhejiang university, Henan People’s hospital ethics committee, ethics committee of the first affiliated hospital of the third military medical college of PLA, ethics committee of the first affiliated hospital of China Medical University, ethics committee of General Hospital of Shenyang military Region, Renji hospital ethics committee, ethics committee of the second affiliated hospital of Guangzhou medical college, ethics committee of General Hospital of Guangzhou military Region. Written informed consent will be obtained from each participant prior to enrollment. The trial is registered on the Chinese Clinical Trial Registry: ChiCTR-TRC-13003127.

### Monitor and supervise of the trial

The whole process of the trial will be supervised by a Trial Steering Committee and a data and safety monitoring committee. The Trial Steering Committee consists of 3 independent members, they will guide and supervise the trial from the beginning, and provide expert advice to the investigators on all aspects of the trial, including decisions about continuation or termination of the trial or amendments to the protocol.

The data and safety monitoring committee consists of 2 biostatisticians, 2 professor expertise in the treatment of intracranial aneurysms, and an independent company. They will work together to make sure that monitor and supervise the whole process of the trial to ensure that 1)the trial is being conducted in accordance with the principles of Good Clinical Practice and the relevant regulations of China. 2) any adverse events during the study are promptly reported to the sponsor, the Clinical Trials Units, and related agency. 3) any variances from the clinical trial program to the sponsor. 4) the devices are used as suggested by the protocol. 5) all subjects are fully informed, and informed consent are achieved. 6) the clinical report forms are promptly and clearly completed. And they will review the progress of the trial and accruing trial data, including updated figures on recruitment, data quality and safety data, and provide expert advice to the investigators on all aspects of the trial, including decisions about continuation or termination of the trial or amendments to the protocol.

## Discussion

Large and giant aneurysms carry the risk of a significantly worse outcome compared with small aneurysms [3]. The ISUIA study showed that the risks of rupture within 6 years for large and giant aneurysms are as high as 13% and 27%, respectively [2]. The treatment of this entity, whether with conventional endovascular treatment or surgery, imposes a great challenge for both neurosurgeons and neurointerventionalists. A deconstructive approach such as internal carotid artery(ICA) occlusion requires sufficient compensation from other vessels; even so, rates of ischemia as high as 4%-15% have been recorded [7]. In addition, there are also concerns about de novo aneurysm following ICA occlusion [31]. Bypass surgery, especially high-flow bypass, preceding ICA occlusion may help reduce ischemic complications, but operative complications are not uncommon, though reports in different manuscripts vary [32]. In contrast, reconstructive approaches such as stent-assisted coiling aiming to preserve the parent artery are associated with much higher recanalization rates, ranging from 19.2% to 50%. However, the use of FDs, including Pipeline, Silk, and Tubridge, have recently shown excellent and promising results [9-14,16]. Because of this, we began this prospective trial, which may provide more information on the safety and efficacy of the Tubridge flow diverter and may change the treatment strategy for large or giant aneurysms if the hypotheses are confirmed.

A similar trial, MARCO POLO, which was designed to compare the results of coiling (with balloon remodeling and/or stents when necessary) to those of Silk FD implantation, is being conducted in 7 countries around the world [33]. However, there are apparent differences between our trial and MARCO POLO, such as the target aneurysms and the different active comparators used.

Although sporadic studies have reported FD application in ruptured intracranial aneurysms, basilar trunk aneurysms, and aneurysms beyond the circle of Willis, its safety is not confirmed yet [34-37]. Anti-platelet therapy may enhance the aneurysm rupture risk. Furthermore, ischemic events may also be encountered more often when FDs are implanted at the basilar trunk or in parent arteries beyond the circle of Willis due to the involvement of small perforators. To ensure the maximum safety of all subjects and to obtain results more efficiently, we restricted our target to unruptured Internal carotid artery or vertebral artery aneurysms.

The Enterprise is a type of intracranial self-expansion stent with a closed-cell design. It is one of the most widely used stents in clinics around the world [28,38,39], and sufficient data has also demonstrated its safety. In this trial, therefore, we choose to use Enterprise-assisted coiling in the comparative arm of the study to evaluate the safety and efficacy of the Tubridge FD.

### Trial status

The trial was initiated in October 2012 and is currently open for recruitment.

## Competing interests

During the development, Prof. Liu had given some advice about the design of this device, and offered some data about our previous hemodynamic studies. The other authors declare they have no competing interests.

## Authors’ contributions

QHH and JML conceived of the study. YX, BH, WYZ, and YBF designed the study. PFY and YZ contributed to the draft of the manuscript. QL and RZ contributed to the revision of the manuscript. All authors read and approved the final manuscript.

## Pre-publication history

The pre-publication history for this paper can be accessed here:

http://www.biomedcentral.com/1471-2377/14/97/prepub
